# Waterborne *Elizabethkingia meningoseptica* in Adult Critical Care^1^

**DOI:** 10.3201/eid2201.150139

**Published:** 2016-01

**Authors:** Luke S.P. Moore, Daniel S. Owens, Annette Jepson, Jane F. Turton, Simon Ashworth, Hugo Donaldson, Alison H. Holmes

**Affiliations:** Imperial College Healthcare NHS Trust, London, UK (L.S.P. Moore, A. Jepson, S. Ashworth, H. Donaldson, A.H. Holmes);; Imperial College London, London. (L.S.P. Moore, A.H. Holmes);; St. George’s Healthcare NHS Trust, London (D.S. Owens);; Public Health England, London (J.F. Turton)

**Keywords:** Matrix-assisted laser desorption/ionization time-of-flight, intensive care, antimicrobial drug resistance, water, Chryseobacterium meningosepticum, Flavobacterium meningosepticum, Elizabethkingia meningoseptica, United Kingdom, adults

## Abstract

*Elizabethkingia meningoseptica* is an infrequent colonizer of the respiratory tract; its pathogenicity is uncertain. In the context of a 22-month outbreak of *E. meningoseptica* acquisition affecting 30 patients in a London, UK, critical care unit (3% attack rate) we derived a measure of attributable morbidity and determined whether *E. meningoseptica* is an emerging nosocomial pathogen. We found monomicrobial *E. meningoseptica* acquisition (n = 13) to have an attributable morbidity rate of 54% (systemic inflammatory response syndrome >2, rising C-reactive protein, new radiographic changes), suggesting that *E. meningoseptica* is a pathogen. Epidemiologic and molecular evidence showed acquisition was water-source–associated in critical care but identified numerous other *E. meningoseptica* strains, indicating more widespread distribution than previously considered. Analysis of changes in gram-negative speciation rates across a wider London hospital network suggests this outbreak, and possibly other recently reported outbreaks, might reflect improved diagnostics and that *E. meningoseptica* thus is a pseudo-emerging pathogen.

## Abstract 2

This outbreak might reflect improved diagnostic testing, indicating that *E. meningoseptica* is a pseudo-emerging pathogen.

*Elizabethkingia meningoseptica* (formerly *Flavobacterium meningosepticum* and, during 1994–2005 *Chryseobacterium meningosepticum*) (*1*) is a gram-negative nonfermenting obligate aerobe. It is widely distributed in the environment (*2*), yet also an acknowledged opportunistic human pathogen. Most frequently associated with neonatal meningitis (*3*,*4*), the organism also has been described in osteomyelitis (*5*) and skin structure infections (*6*,*7*). In addition, *E. meningoseptica* has been associated with colonization of the respiratory tract in ventilated adult patients, but causation of ventilator-associated pneumonia in this cohort is less clear; some studies have attributed pathogenicity (*8*–*10*), but others have found no attributable disease from colonization (*11*,*12*). Outbreaks have been linked to hospital water sources in adult critical care units (*8*,*13*); these outbreaks have been suggested to be attributable to the tolerance exhibited by *Elizabethkingia* species to such environments.

Challenges in the laboratory diagnosis of this organism complicate a true understanding of its role in disease. Difficulties in culture, including variable (strain-dependent) growth on MacConkey agar (*1*) and misidentification on some automated laboratory platforms (*4*,*7*), contribute to diagnostic challenges. Recent changes to clinical laboratory practice, particularly the widespread adoption of matrix-assisted laser desorption/ionization time-of-flight (MALDI-TOF) mass spectrometry, has improved confidence in identification of nonfermenting gram-negative organisms (*14*) and specifically facilitated rapid identification of *E. meningoseptica* from patient samples (*15*). How this advance confounds the reported epidemiology of this organism remains unclear and contributes to the lack of clarity around attributable illness. In the context of recent increased international reporting of *E. meningoseptica* outbreaks among adults, including in the United States (*10*), Brazil (*16*), South Asia (*17*), and Southeast Asia (*8*,*18*), establishing whether *E. meningoseptica* is an emerging pathogenic organism is essential.

We report a retrospective observational study detailing an outbreak of *E. meningoseptica* acquisition in a London teaching hospital adult critical care unit in accordance with the ORION protocol (*19*), analyzing the clinico-physiologic response of patients who acquired *E. meningoseptica*, and deriving a measure of attributable illness. We analyzed case identification in the context of the wider changes to diagnostic laboratory practice to determine whether *E. meningoseptica* is an emerging, or pseudo-emerging (i.e., previously present but unidentified or underidentified), organism (*20*).

## Materials and Methods

### Setting

The outbreak occurred in a 16-bed critical care unit in a West London teaching hospital that receives acute medicine, acute surgery, tertiary referral vascular surgery, and major trauma patients from a 400-bed London teaching hospital. The hospital is part of a wider 5-hospital network across West London with overarching institutional policies, including for infection prevention and control and antimicrobial stewardship. The critical care unit comprises 8 en suite single rooms (each with a room sink and a lobby sink) and two 4-bed bays (with 1 clinical sink per bed). Infrared taps are used in all clinical sinks. An off-site sterilization facility processes all endoscopes and procedural equipment. Critical care staffing levels meet mandatory requirements, and a multidisciplinary infection control team provides support with daily critical care antimicrobial rounds. A critical care resistant-organism screening program is in place, and all patients admitted for critical care have cross-infection screening comprising methicillin-resistant *Staphylococcus aureus* sampling (nasal and groin) at admission and then weekly, and resistant gram-negative organism sampling (rectum and throat) once per week.

### Microbiological Investigation

A centralized microbiology laboratory processes samples from the 5-hospital network in accordance with standard UK laboratory operating procedures (*21*). Specifically, cross-infection sample processing occurs in line with detection of extended-spectrum β-lactamase/carbapenem-hydrolyzing organism protocols (*21*). Blood cultures are incubated by using a BACTEC system (Becton Dickinson, Franklin Lakes, NJ, USA). Since June 2011, organisms have been identified by Biotyper MALDI-TOF mass spectroscopy (Bruker Daltonik GmbH, Bremen, Germany) with previously described methods used for identifying nonfermenting gram-negative organisms (*14*,*22*); previously, identification was by API (bioMérieux, Marcy l’Etoile, France). Susceptibility testing is by disk diffusion using British Society of Antimicrobial Chemotherapy methods and interpretative criteria (*23*). A representative of the outbreak strain underwent MIC determination by using agar dilution for a broad range of antimicrobial agents at a national reference laboratory (*24*). 

Water from all clinical taps in the critical care unit was sampled for bacterial colonization in July 2012, July 2013, and December 2013. A total of 100 mL of water was collected from each tap, filtered by using a 0.45-μ filter membrane, and incubated on MacConkey agar in air at 37°C for 48 hours. Oxidase-positive non–lactose-fermenting colonies were subcultured onto nutrient agar and a 10-μg meropenem disk placed on the inoculum. Organisms displaying meropenem resistance were further identified by using MALDI-TOF mass spectrometry. Clinical and environmental isolates were compared by using pulsed-field gel electrophoresis (PFGE) of XbaI-digested isolate genomic DNA as previously described (*25*), except that switch times of 1–25 seconds were used.

### Cases

The index case was identified on January 12, 2012, in a patient from whom *E. meningoseptica* was grown from a respiratory tract sample. This patient and all those in whom *E. meningoseptica* was subsequently isolated from clinical or screening samples were defined as case-patients and are analyzed here. During January 2012–October 2013, we identified 30 cases from among 983 new patients admitted to the critical care unit.

### Determination of Attributable Illness

All case-patients had retrospective interrogation of their electronic critical care records to determine clinico-physiologic parameters (pulse rate, oxygen requirements, temperature, C-reactive protein [CRP], leukocyte count, chest radiography); primary outcomes (discharge from critical care, death during admission); and antimicrobial history. The microbiology information management system was interrogated to identify all relevant isolates in the 7 days before or after acquisition, to which any evident clinical infection could otherwise be attributed. After excluding patients in whom multiple organisms were identified, we were able to identify case-patients with monomicrobial *E. meningoseptica* acquisition and, in this subgroup, analyze the trend in clinico-physiologic parameters in the 48 hours before and after acquisition. Three systemic inflammatory response syndrome (SIRS) parameters were investigated (because most patients were ventilated, respiratory rate as a parameter was excluded): new temperature change to <36°C or >38°C, new increase in pulse rate to >90 beats per minute, and new change in leukocyte count to <4 >12 × 10^9^ cells/L. We investigated 3 additional criteria: new rise in fraction of inspired oxygen requirement >0.1, new CRP >100 mg/L, and new pulmonary infiltrates on plain chest radiography.

### Outbreak Investigation

We undertook spatiotemporal analysis of cases by correlating bed occupancy of confirmed case-patients against each other and possible environmental reservoirs to identify possible routes of cross-transmission or point sources. This analysis was reviewed against sequential interventions to determine effectiveness in outbreak curtailment. Data from serial routine 6-monthly antimicrobial use point-prevalence studies (conducted across the hospital network) were analyzed to identify trends in antimicrobial use. We also analyzed the microbiology information management system to identify any other *E. meningoseptica* in the wider 5-hospital network during the outbreak period and for the 2 preceding years. This analysis enabled identification of any possible out-of-cohort secondary cases and enabled a wider analysis of the epidemiology of *E. meningoseptica* within the hospital network. Ethical approval was not required for this study; outbreak investigation and analysis was classed as service evaluation by the head of regulatory compliance at the host institute.

## Results

We identified 30 patients as acquiring *E. meningoseptica* during the outbreak, yielding an attack rate of 3% for patients admitted to critical care. The median age of *E. meningoseptica* case-patients was 45 years (range 17–83 years); 73% were male (Table 1), compared with a critical care all-admission median age of 55 years (range 8–95 years) and 68% male. Before *E. meningoseptica* acquisition, the median time spent in the critical care unit was 17 days (range 4–35 days), and 26 patients had received broad-spectrum antimicrobial drug regimes (piperacillin/tazobactam or meropenem) in the week preceding acquisition. Of the 30 patients in whom *E. meningoseptica* was identified, 24 had the organism isolated from specimens taken for a clinical indication; for 6, the organism was isolated only through routine screening.

**Table 1:** Clinical and epidemiologic patient characteristics from an *Elizabethkingia meningoseptica* outbreak in an adult critical care unit, West London, UK, 2012–2013*

Patient no.	Age, y/sex	Admission category	Date of *E. m.* acquisition	Hospital day of acquisition	Sample type†	Antimicrobial therapy immediately before *E. m.* acquisition	*E. m.* treatment regimen	Clinical outcome	PFGE designation
1	29/M	Trauma	2012 Jan 12	35	Respiratory	None	None	Discharged	NA
2	45/F	Medical	2012 Feb 27	9	Respiratory	TZP	None	Discharged	EZ1
3	58/M	Medical	2012 Mar 2	22	Respiratory	MEM + CAS	None	Discharged	EZ1
4	34/M	Trauma	2012 Mar 10	18	Respiratory	TZP	None	Discharged	NA
5‡	28/M	Trauma	2012 Mar 20	15	Screening	MEM	TGC	Discharged	EZ2
6	64/M	Surgical	2012 Mar 22	4	Respiratory	None	None	Discharged	NA
7‡	77/M	Medical	2012 Mar 28	10	Screening	MEM + MTZ	None	Discharged	EZ1
8	69/M	Trauma	2012 Apr 18	11	Screening	MEM	None	Discharged	NA
9‡	35/F	Trauma	2012 May 21	19	Screening	TZP + AFG	TMP/SXT	Discharged	EZ2
10‡§	35/F	Surgical	2012 Jul 16	14	Respiratory	MEM	TMP/SXT	Discharged	NA
11‡§	60/F	Medical	2012 Jul 21	22	Respiratory	None	None	Died	EZ1
12	55/M	Surgical	2012 Jul 27	14	Respiratory	TZP + VAN	None	Died	EZ1
13	43/M	Trauma	2012 Sep 13	6	Screening	MEM + MTZ	None	Discharged	NA
14	40/M	Trauma	2012 Dec 27	13	Respiratory	MEM + VAN	None	Discharged	NA
15	40/F	Medical	2013 Jan 3	31	Blood culture	MEM + MTZ	TGC	Died	NA
16‡	23/M	Trauma	2013 Jan 14	13	Respiratory	None	None	Discharged	EZ1
17	57/M	Trauma	2013 Jan 14	13	Respiratory	TZP + FCA	None	Discharged	NA
18‡§	19/M	Trauma	2013 Mar 26	25	Respiratory	TZP + MTZ	None	Discharged	NA
19‡	70/M	Vascular	2013 Apr 8	11	Respiratory	MEM + FCA	TGC	Discharged	Unique
20‡§	61/F	Trauma	2013 Apr 27	11	Respiratory	MEM	TGC	Died	Unique
21‡§	43/M	Surgical	2013 May 1	12	Respiratory	MEM +AFG	None	Discharged	NA
22‡	17/M	Trauma	2013 May 22	28	Screening	MEM + MTZ	None	Discharged	EZ3
23§	60/M	Medical	2013 May 30	13	Respiratory	TZP + FCA	None	Died	NA
24‡§	75/F	Trauma	2013 Jun 21	13	Respiratory	TZP	TGC	Discharged	NA
25	75/M	Trauma	2013 Jun 22	12	Respiratory	MEM + VAN	None	Discharged	NA
26	77/F	Medical	2013 Aug 2	22	Respiratory	TZP	TMP/SXT	Discharged	EZ1
27	31/M	Trauma	2013 Sep 15	26	Respiratory	MEM + VAN	TGC	Discharged	NA
28‡§	83/M	Surgical	2013 Sep 15	28	Respiratory	TZP + FCA	TMP/SXT	Discharged	NA
29‡§	32/M	Trauma	2013 Oct 10	11	Respiratory	TZP + VAN	TMP/SXT	Discharged	NA
30	48/M	Trauma	2013 Oct 29	34	Respiratory	TZP + VAN	None	Discharged	NA
31¶	34/F	Trauma	2014 Apr 12	1	Screening	None	None	Discharged	Unique

*AFG, anidulofungin; CAS, caspofungin; Dis, discharged; *E. m.*, *E. meningoseptica*; FCA, fluconazole; MEM, meropenem; MTZ, metronidazole; NA, isolate unrecoverable for PFGE analysis; PFGE, pulsed-field gel electrophoresis; TGC, tigecycline; TMP/SXT, trimethoprim/sulfamethoxazole; TZP, piperacillin/tazobactam; VAN, vancomycin; TGC, tigecycline; AFG, anidulofungin. †Respiratory sample types included nondirected bronchoalveolar lavage or endotracheal suction. Cross-infection screens comprise throat, rectum, nose, and groin swab specimens. ‡Patients in whom no other pathogen was identified in the 7 days before or after isolation of *E. meningoseptica*. §Patients in whom chest radiography demonstrated new-onset signs consistent with a pneumonic process in the 48 hours before and after *E. meningoseptica* isolation. ¶Postoutbreak infection.

### Microbiological Investigation

Identification of isolates from patients and water samples by MALDI-TOF mass spectrometry gave spectra concordant with *E. meningoseptica* for all isolates with relative intensity of matched peak scores >2.1. Disk diffusion susceptibility testing demonstrated in vitro resistance to amoxicillin, amoxicillin/clavulanic acid, temocillin, cefuroxime, cefotaxime, ceftazidime, ertapenem, meropenem, gentamicin, tobramycin, amikacin, and colistin but susceptibility to ciprofloxacin, piperacillin/tazobactam, tigecycline, and trimethoprim/sulfamethoxazole. The antibiograms were consistent for isolates from all 30 patients; MICs of selected agents for a representative isolate are shown in Table 2.

**Table 2:** MICs of selected antimicrobial agents tested against a representative isolate from an *Elizabethkingia meningoseptica* outbreak strain from an adult critical care unit, West London, UK, 2012–2013*

Antimicrobial agent	MIC, mg/L	Interpretation
Ceftazidime	256	Nonsusceptible
Piperacillin/tazobactam	16	Susceptible
Meropenem	>32	Nonsusceptible
Imipenem	64	Nonsusceptible
Aztreonam	>64	Nonsusceptible
Gentamicin	16	Nonsusceptible
Tobramycin	>32	Nonsusceptible
Amikacin	32	Nonsusceptible
Colistin	>32	Nonsusceptible
Ciprofloxacin	1	Intermediate
Minocycline	0.5	Unknown
Trimethoprim/sulfamethoxazole	0.25	Susceptible

*MICs were determined by serial agar dilution by using established methods (*24*). Interpretation of MICs used established British Society of Chemotherapy breakpoints. The intrinsic metallo- and extended-spectrum-β-lactamases exhibited by *E. meningoseptica* mean the apparent in vitro susceptibility of the organism to piperacillin/tazobactam should be viewed with caution.

In addition to the isolates derived from patients, 7 *E. meningoseptica* isolates were identified from 5 sinks (1 in July 2012 when 2 additional taps were identified to have *Pseudomonas* spp. colonization; 4 in July 2013 when no further taps had *Pseudomonas* spp. colonization; no organisms were identified in December 2013). Routine analysis of bronchoscope rinse water from decontamination units during the investigation period showed no growth.

**Figure 1:**
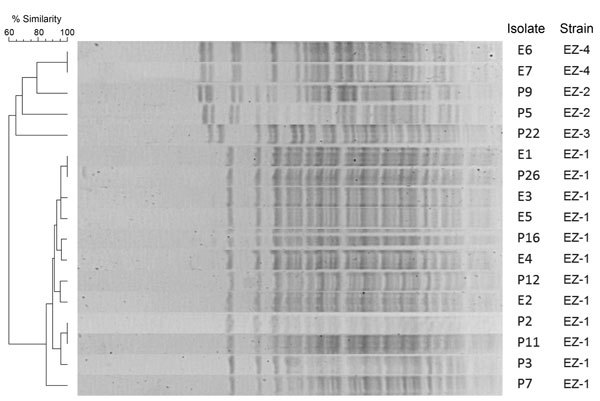
Pulsed-field gel electrophoresis profiles of *XbaI*-digested genomic DNA from patient (P) and environmental (E) *Elizabethkingia meningoseptica* isolates from an outbreak in an adult critical care unit, London, UK, 2012–2013. Two additional isolates from patients demonstrated unique pulsed-field gel electrophoresis profiles and are not shown. Patient numbers (e.g., P9) match those given in Table 1.

PFGE typing (Figure 1) showed that of the 12 patient isolates retrievable, 7 shared a common PFGE pattern (denoted EZ1), 2 shared a different profile (EZ2), 1 had a further identifiable profile (EZ3), and 2 others had unique profiles. Comparative PFGE typing of the 7 environmental isolates demonstrated that 5 were indistinguishable from the EZ1 outbreak strain; the remaining 2 isolates shared a PFGE pattern not identified among patient isolates (EZ4). The 5 EZ1 environmental isolates were isolated from taps from 3 different sink units in the critical care unit.

### Attributable Illness

**Figure 2:**
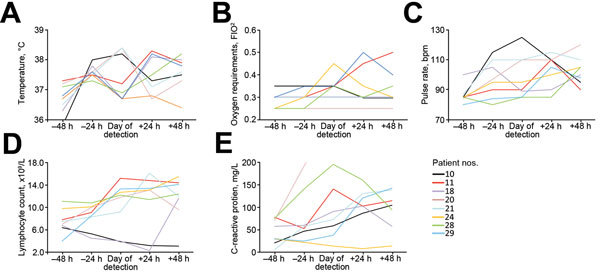
Clinicophysiologic parameters of patients with monomicrobial acquisition of *Elizabethkingia meningoseptica* in an outbreak in an adult critical care unit, London, UK, 2012–2013. Thirteen patients in the outbreak cohort were identified as having monomicrobial *E. meningoseptica* acquisition. Of these, 8 patients demonstrated an increase in 5 clinicophysiologic parameters of inflammation during the 48 hours before and after acquisition of *E. meningoseptica*: A) body temperature; B) oxygen saturation; C) pulse rate; D) lymphocyte count; and D) C-reactive protein. Patient numbers match those given in Table 1.

Eleven of the 30 case-patients received antimicrobial drug therapy targeted at *E. meningoseptica*, in all cases for a clinical diagnosis of hospital-acquired pneumonia. Thirteen patients were identified within the outbreak cohort in whom no discernible microbiological evidence of other pathogens was found in the 7 days before or after *E. meningoseptica* acquisition (Figure 2). In the 48 hours before and after *E. meningoseptica* acquisition, in terms of SIRS response, 7 case-patients had new-onset fever, 7 had new tachycardia, and 8 had new leukocyte count change. Additionally, 4 had increasing oxygen requirements, 7 had new increase in CRP, and 8 had new infiltrates on chest radiography. Moreover, targeted *E. meningoseptica* antimicrobial therapy was begun on 8 of these patients by the physicians coordinating care. Therefore, attributable illness (SIRS >2) from acquisition of *E. meningoseptica* in this outbreak was 54%.

Five case-patients died, including 2 of those deemed to have monomicrobial *E. meningoseptica* acquisition. However, the cause of death in those 2 patients was not due to infection; that is, no deaths were attributed to *E. meningoseptica* acquisition in this outbreak.

### Outbreak Investigation

Analysis of bed occupancy demonstrated that for most of the time the critical care unit had contemporaneous case-patients present. However, 2 notable periods where no cases were identified (October 2012–December 2013 and January–March 2013) suggested a point source was more likely than person-to-person transmission in perpetuating the outbreak. Spatial correlation was observed between all colonized patients and environmental isolates in 1 quadrant of the critical care unit (2 side rooms and 1 bay); environmental sampling implicated 3 clinical sinks as the point source in this quadrant. No ongoing building or plumbing work elsewhere in the contiguous water system was identified.

Analysis of the antimicrobial point-prevalence studies showed that 63%–79% of all patients in the critical care unit were receiving antimicrobial drugs at any 1 time, but no directional trend was exhibited. Antimicrobial drug use in the outbreak unit demonstrated no major difference from that in the other critical care units in the hospital network.

### Estimating the Effect of MALDI-TOF Mass Spectrometry Introduction on Identification of *E. meningoseptica*

Interrogation of the microbiology information management system identified 8 other *E. meningoseptica* isolates throughout the wider hospital network: 1 patient in the study hospital in March 2013 for whom no connection to the critical care unit could be established, and 7 patients in the 4 other hospitals in the network during January 2010–October 2013. None of these were from critical care units, and no discernible health care contact was found among the case-patients. Only 2 of these 8 additional cases were detected before MALDI-TOF mass spectrometry was introduced into routine laboratory practice in June 2011, meaning 6 (and all 30 of the outbreak case-patients) were identified after its introduction. Further analysis of the microbiology information management system revealed that throughout the hospital network during January 2010–June 2011, a total of 17% of non–lactose-fermenting gram-negative organisms were not identified to genus/species level; after introduction of the MALDI-TOF mass spectrometry, during July 2011–October 2013, this percentage decreased to 10.9%.

### Water Reservoirs and Control

Interventions to attempt containment of the outbreak included (sequentially): domestic process review (single cloth per sink; “clean-to-dirty” cleaning protocol) and decluttering of clinical areas (August 2012); instigation of daily sink trap chlorination in all clinical sinks (August 2012); exchange of clinical sink traps (September 2012); and water course remodeling, including removal of flexible tubing segments (September–December 2012). Use of alcohol gel after hand washing was advocated throughout the outbreak. These steps failed to control the outbreak; however, after initiation of 3 times per day automated flushing of all clinical tap units in October 2013, water testing in December 2013 demonstrated an absence of *E. meningoseptica* or *Pseudomonas* species, and no further isolates were identified from patients in the critical care unit from November 2013 onward. The exception was 1 isolate from a cross-infection sample in a patient admitted in April 2014, detected from screening samples taken on the day of admission; typing of this organism showed a unique PFGE profile not related to any of the previously identified isolates.

## Discussion

In the context of a prolonged outbreak of *E. meningoseptica* acquisition in an adult critical care unit of a London teaching hospital, we found that acquisition of this organism was associated with clinically significant attributable illness in approximately half of patients, evidence against this organism being a nonpathogenic colonizer. We found clinical and molecular epidemiologic evidence indicating acquisition is associated with water sources in the critical care unit; however, within these water samples we also identified numerous varied strains of *E. meningoseptica*, suggesting more widespread dissemination of this organism than previously thought. From our analysis of microbiology data throughout the hospital network, we found a marked excess of identified *E. meningoseptica* (both outbreak and nonoutbreak) and a contemporaneous decrease in unspeciated nonfermenting gram-negative organisms after MALDI-TOF mass spectrometry was introduced. We propose that wider introduction of this technology across clinical laboratories might be overcoming previous difficulties in identifying *E. meningoseptica*, possibly contributing to the recent increase in reported outbreaks of this emerging pathogen (*8*,*10*,*16*–*18*).

New-onset rise in temperature, tachycardia, and inflammatory markers occurred in half of the patients who acquired *E. meningoseptica* and culminated in clinical decisions to instigate targeted therapy in the absence of any other organisms. This finding suggests *E. meningoseptica* causes clinical infection and does not just colonize patients in critical care. Furthermore, the high frequency of isolation of *E. meningoseptica* from respiratory samples across the outbreak cohort, combined with new-onset radiographic changes in half of patients with monomicrobial *E. meningoseptica*, suggests that this pathogen is a cause of hospital-acquired pneumonia. Biological plausibility exists, with virulence factors including a propensity for biofilm formation (*26*,*27*), intracellular invasion (*28*), and chromosomal (*29*) and plasmid (*30*) mediated resistance to many antimicrobial drugs, including commonly used β-lactams. This marked antimicrobial drug resistance has been previously documented to include 3 *bla*_CME_ genes coding for extended-spectrum serine-β-lactamase (Ambler class D) and 2 unrelated metallo-β-lactamases conferring carbapenem resistance: *bla*_B_ (subclass B1) and *bla*_GOB_ (subclass B3) (*31*). Acquisition of further resistance elements, including *bla*_KPC_, also has been documented (*32*). Phenotypic susceptibility testing on the isolates from this outbreak supports such a marked resistance phenotype, particularly to β-lactam antimicrobial drugs. This high level of antimicrobial resistance may have accounted for the excess appearance of the organism in patients who had a history of broad-spectrum antimicrobial drug therapy; 87% of the patients who acquired *E. meningoseptica* had a history of preceding antimicrobial use (predominantly piperacillin/tazobactam and meropenem), compared with a background of 63%–79% among nonoutbreak critical care patients. Drug resistance also led to a limited armamentarium with which to treat; whereas our treatment strategies were susceptibility testing driven (trimethoprim/sulfamethoxazole and tigecycline), other agents have been advocated, including some typically considered to target gram-positive organisms (*3*).

The noted potential for *E. meningoseptica* to display a strong biofilm biotype might also explain the failure of many of the infection control interventions during this outbreak. The failure of chlorine has been documented (*33*), but use of post–hand washing alcohol gel, previously found effective in terminating outbreaks (*13*,*34*), was not effective in our experience. The apparent success of regular sink flushing in terminating our outbreak might be attributed to the sheer force exerted during this process and is advocated in recent UK guidance for augmented care areas where waterborne pseudomonads are of concern (*35*). The return of the organism in a single patient in April 2014, seven months after the proposed outbreak termination, might be attributable to a failure in the automated flushing protocols but more likely represents contamination from a sink in a nearby area of the hospital (i.e., operating rooms) that does not practice the auto-flushing protocol or from outside the health care environment. Biofilm formation also might account for the observed predilection for respiratory tract acquisition, and we speculate that, in addition to antimicrobial drug therapy, in those with airway adjuncts repeated device changes might be helpful. Small-molecule disruption of biofilms might in the future provide an alternative therapeutic avenue (*36*).

The identification of numerous strains (albeit with 1 predominating) of *E. meningoseptica* in patients and in water sources suggests a wider issue in the water microbiome. The historical difficulties in identifying *E. meningoseptica* from other nonfermenting gram-negative organisms (including *Pseudomonas* species) in both patient and environmental samples mean that the advent of MALDI-TOF mass spectrometry might simply be helping to describe *E. meningoseptica* epidemiology, and the recent increase in reported outbreaks might indicate ascertainment bias. This possible bias is supported by the wider microbiology data, with few *E. meningoseptica* isolates being identified anywhere in the hospital network before introduction of MALDI-TOF mass spectrometry, after which not only were many more identified, but a concomitant fall in the frequency of nonidentified gram-negative organisms also was observed. Although MALDI-TOF mass spectrometry might therefore be aiding the early phase of outbreak detection through improved organism identification, the extent to which this organism represents an emerging pathogen, as opposed to how much preexisted and is simply newly identified, is unclear. Further work on the utility of MALDI-TOF mass spectrometry in outbreak detection and investigation is warranted, and an additional role in typing might be feasible (*37*–*40*). Integration of this platform into clinical practice, as is happening in many laboratories, must be given due consideration as to such potential unintended consequences.

A failure to subculture many of the isolates from the cohort for PFGE typing is a noted limitation of this study. As described, however, variable growth on commonly used media is a feature of this organism. Moreover, the typing that was conducted was hardly circumstantial and was sufficient to demonstrate a link between isolates from water sources and from patients. A further limitation of this study, in delineating the attributable illness, was the low number of patients for whom clinico-physiologic parameters were analyzed. However, inclusion of cases was purposefully strict, limiting cases to persons from whom no organisms other than *E. meningoseptica* were isolated. This restriction was to enable changes in clinico-pathologic variables to be specifically associated with *E. meningoseptica* rather than any co-cultured organisms; however, the possibility remains that other organisms were present and not cultured.

Transmission of waterborne *E. meningoseptica* to adult critical care patients has an attributable illness rate of 54%. Advances in rapidity and accuracy of microbiology diagnostics, including through adoption of MALDI-TOF mass spectrometry, is leading to increased detection of this organism providing an improved understanding of critical care clinical infections and the waterborne hospital microbiome. Consequently, the recent international increase in *E. meningoseptica* outbreaks in adults, including from the United States, Brazil, and South and Southeast Asia, might indicate a pseudo-emerging, rather than an emerging, nosocomial pathogen. Further work is needed, and network analysis and whole-genome sequencing are likely to facilitate greater understanding of the wider transmission potential of *E. meningoseptica*. Given the attributable illness, the organism’s marked antimicrobial resistance profile, and its endurance against standard infection prevention and control procedures, development of robust interventions to combat waterborne outbreaks of this pathogen among critically ill adults is urgently needed.
